# Proteomics in Deaths by Drowning: Diagnostic Efficacy of Apolipoprotein A1 and α-1 Antitrypsin, Pilot Study

**DOI:** 10.3390/diagnostics10100747

**Published:** 2020-09-24

**Authors:** Diana Hernández-Romero, Encarnación Sánchez-Rodríguez, Eduardo Osuna, Agustín Sibón, Miriam Martínez-Villanueva, José A. Noguera-Velasco, María D. Pérez-Cárceles

**Affiliations:** 1 Department of Legal and Forensic Medicine, Biomedical Research Institute (IMIB), Regional Campus of International Excellence “Campus Mare Nostrum”, Faculty of Medicine, University of Murcia, 30100 Murcia, Spain; eosuna@um.es (E.O.); mdperez@um.es (M.D.P.-C.); 2 Institute of Legal Medicine and Forensic Science, 11071 Cádiz, Spain; nitamed.s@hotmail.com (E.S.-R.); agustin.sibon.ius@juntadeandalucia.es (A.S.); 3 Clinical Analysis Service, Hospital University “Virgen de la Arrixaca”, 30100 Murcia, Spain; miriam.marti@gmail.com (M.M.-V.); josea.noguera@gmail.com (J.A.N.-V.)

**Keywords:** proteomics, drowning, forensic diagnosis, apolipoprotein A1, α-1 antitrypsin

## Abstract

Drowning is one of the leading causes of death worldwide. The pathophysiology of drowning is complex and, sometimes, interpretation of the circumstances of death in the autopsy becomes the main source of information in its diagnosis. New advances in medical research, such as proteomics, especially in forensic pathology, are still in the development. We proposed to investigate the application of Mass Spectrometry-based technologies, to identify differentially expressed proteins that may act as potential biomarkers in the postmortem diagnosis of drowning. We performed a pilot proteomic experiment with the inclusion of two drowned and two control forensic cases. After applying restrictive parameters, we identified apolipoprotein A1 (ApoA1) and α-1 antitrypsin as differentially expressed between the two diagnostic groups. A validation experiment, with the determination of both proteins in 25 forensic cases (16 drowned and 9 controls) was performed, and we corroborated ApoA1 higher values in the drowning group, whereas α-1 antitrypsin showed lower levels. After adjusting by confounder factors, both remained as predictive independent factors for diagnosis of drowning (*p* = 0.010 and *p* = 0.022, respectively). We constructed ROC curves for biomarkers’ levels attending at the origin of death and established an ApoA1 cut-off point of 100 mg/dL. Correct classification based on the diagnosis criteria was reached for 73.9% of the cases in a discriminant analysis. We propose apolipoprotein A1 (with our cutoff value for correct classification) and α-1 antitrypsin as valuable biomarkers of drowning. Our study, based on forensic cases, reveals our proteomic approach as a new complementary tool in the forensic diagnosis of drowning and, perhaps, in clinical future implications in drowned patients. However, this is a pilot approach, and future studies are necessary to consolidate our promising preliminary data.

## 1. Introduction

Drowning is the leading cause of death among young males. Worldwide, drowning accounts for an estimated 360,000 deaths annually, representing 7% of all injury-related fatalities [1]. As an etiology of violent death, drowning has increased worldwide in recent years, particularly as an accidental etiology related with aquatic activities [2]. Different studies revealed an increase in the proportion of drowning associated with natural water-related catastrophes (floods, tropical storms, hurricanes and even tsunamis) [3,4]. Following extensive discussion and debate, the World Health Organization agreed on the following definition: “Drowning is the process of experiencing respiratory impairment from submersion/immersion in liquid [5]”. The estimation of the full impact of drowning deaths and disability due to drowning are grossly underreported, especially in countries with low income that have limited resources to collect data [6]. For every person who dies from drowning, another four persons receive care in the emergency department for nonfatal drowning [7].

In water-related deaths involving submersion, it becomes necessary to investigate how and why the body became submerged for the most accurate diagnosis of the cause of death. The postmortem diagnosis of drowning, postmortem interval determination and different characteristics related to the environment continue to be topics of interest, in an effort to improve medico-legal death investigation and the resolution of water-related deaths. Currently, the diagnosis of drowning is based largely on autopsy findings, with the exclusion of other causes of death, interpreted within the context of the death circumstances. Prolonged submersion with decomposition adds another dimension of diagnostic difficulty [8]. It is well-known that drowning is a complex process with several physical reactions, leading to acute respiratory distress syndrome, hypoxic respiratory failure, and cardiorespiratory arrest [8,9].

Recent studies in biochemistry, histology and immunohistochemistry have provided very interesting results on the post-mortem diagnosis of drowning, complementing classical autopsy findings. For instance, useful methods for differential diagnosis between freshwater and saltwater drowning have been reported [10,11,12,13,14]. Novel technologies such as proteomics are still in the stage of exploration in forensic pathology [15], and mainly focused to determine PMI [16,17,18]. We proposed to investigate the usefulness of MS-based spectrometry to identify biomarkers that add relevant information in the postmortem diagnosis of drowning and the circumstances of the death. To our knowledge, no studies have been performed within the context of the proteomic application to the diagnosis of death by drowning. In addition, investigating the proteomic profile in forensic cases may be a valuable tool to improve our knowledge of the pathophysiology of drowning, information that can be key in the clinical diagnosis and treatment of hospitalized drowning patients.

## 2. Materials and Methods

### 2.1. Study Design

Corpses from routine forensic autopsies were selected for inclusion at the Institute of Legal medicine and Forensic Sciences of Cádiz (Spain). The cases were selected randomly from among routine medico-legal autopsies. Exclusion criteria were aspiration of blood or gastric contents and macroscopic or microscopic signs of putrefaction.

To minimize postmortem artifacts, the bodies were refrigerated at 3 (1–4) °C until the autopsy was performed. According to the scene, cause and circumstances of death, together with autopsy findings (external foam, frothy fluid in airways, overlap of the medial edges of the lungs in drowning), cases were classified into two groups: deaths by drowning and deaths by other causes. In cases of drowning histological examination (hematoxylin eosin staining) showed intra-alveolar oedema and dilatation of the alveolar spaces, with secondary compression of the septal capillaries. For our pilot proteomic experiment, we selected 4 cases and divided corpses in two groups, paired by age and gender in deaths by drowning (one man and one woman) and control group with different pathophysiological kind of deaths (one man death by hanging (asphyctic-like background) and one women death by polytrauma).

The study was approved by the regional authority of justice in Andalucía (Spain), and the Research Ethics Committee of the University of Murcia (Approval ID: 1898/2018, approval date: 13-04-2018). Current regulations that guarantee the confidentiality of personal data and its automated treatment were respected.

### 2.2. Procedure for Extraction of Biological Fluids

Plasma samples were obtained during the autopsy: peripheral blood samples were collected with a sterile syringe from the femoral vein. The samples were centrifuged without delay after extraction, and were stored for preservation at a temperature of −80 °C until the biochemical batch analysis was carried out.

### 2.3. Protein Extraction

Protein depletion from blood samples with acetonitrile was performed according to the protocol described by Kay et al. [19]. A total volume of 60 µL of human plasma was depleted with this method. Each aliquot was diluted with 120 µL of water and vortexed to mix. Acetonitrile (270 µL) was added to each aliquot, and the sample was sonicated for 10 min in an ultrasonic bath. The samples were vortexed briefly and then sonicated for a further 10 min. The protein precipitate was pelleted by centrifugation at 12,000× *g* for 10 min at room temperature. The supernatants were pooled in a clean Eppendorf tube and evaporated to dryness in a vacuum concentrator centrifuge without heating. The sample was reconstituted in 75 µL of buffer with 0.2 M Tris-HCl (pH = 8.5), 2% *w*/*v* SDS, 10% *v*/*v* glycerol.

Protein quantification from all samples was performed using the Pierce BCA Protein Assay Kit (Thermo Scientific, Rockford, IL, USA). Sodium dodecyl sulphate–polyacrylamide gel electrophoresis (SDS–PAGE) was performed at room temperature using 10% acrylamide resolving gel and 5% acrylamide stacking gel [20]. Proteins within gel bands were first reduced and alkylated using DTT and iodoacetamide, respectively, and then digested to peptides by trypsin proteomics grade (Sigma-Aldrich, St Louis, MO, USA), as previously described [21]. The tryptic peptides were analyzed by capillary reversed-phase liquid chromatography coupled online with MS/MS. The column, BioBasic-18, 5 μm particles, 300 Å pore size, 0.18 mm ID-30 mm L (Thermo, San Jose, CA, USA), was connected to a Surveyor MS Pump Plus (Thermo, San Jose, CA, USA), and then coupled with an ion trap mass spectrometer (LXQ, Thermo, San Jose, CA, USA). Mobile phase A was 0.1% formic acid in water and B was 0.1% formic acid in methanol. The ion trap MS was operated in a data-dependent MS/MS mode, where the five most abundant peptide molecular ions in every MS scan were sequentially selected for collision-induced dissociation with a normalized collision energy of 34%. Dynamic exclusion was applied to minimize the repeated selection of peptides previously selected for collision-induced dissociation.

### 2.4. Database Searching

All MS/MS samples were analyzed using Sequest (Thermo Fisher Scientific, San Jose, CA, USA; version IseNode in Proteome Discoverer 2.3.0.523 and X! Tandem (version 2017.2.1.4)). Sequest was set up to search uniprot homo-sapiens filtered organism (163,787 entries) after digestion with trypsin enzyme. X! Tandem was searched with a fragment ion mass tolerance of 1.00 Da and a parent ion tolerance of 1.00 Da. Sequest was searched with a fragment ion mass tolerance of 1.2 Da and a parent ion tolerance of 1.5 Da. Carbamidomethyl of cysteine was specified in Sequest and X! Tandem as a fixed modification. In addition, Glu- > pyro-Glu of the *n*-terminus, ammonia-loss of the *n*-terminus, Gln- > pyro-Glu of the *n*-terminus, oxidation of methionine, acetylation of the *n*-terminus and phosphorylation of serine were also specified in X! Tandem and Sequest as variable modifications.

### 2.5. Criteria for Protein

Scaffold (version Scaffold_4.10.0, Proteome Software Inc., Portland, OR, USA) was used to validate MS/MS based peptide and protein identifications. Peptide identifications were accepted if they could be established at greater than 93.0% probability to achieve an FDR less than 1.0%. Peptide Probabilities from X! Tandem and Sequest were assigned by the scaffold local FDR algorithm. Peptide Probabilities from X! Tandem were assigned by the peptide prophet algorithm [22] with Scaffold delta-mass correction. Protein identifications were accepted if high restrictive conditions where accomplished, that is, they could be established at greater than 99.0% probability and contained at least 1 identified peptide. Protein probabilities were assigned by the protein prophet algorithm [23]. Proteins that contained similar peptides and could not be differentiated based on MS/MS analysis alone were grouped to satisfy the principles of parsimony. Proteins sharing significant peptide evidence were grouped into clusters.

### 2.6. Validation Experiment

For the validation experiment, 25 forensic cases were divided into deaths by drowning (*N* = 16) and deaths by other causes (*N* = 9: 4 cardiovascular deaths, 1 hanged, 2 politrauma, 1 stabbed, 1 death from drug overdose). Plasma samples were analyzed at the Clinical Biochemistry Service of the Hospital University “Virgen de la Arrixaca” to quantify ApoA1 and α-1 antitrypsin levels by immunonephelometry in a BN ProSpec equipment from Siemens Healthcare Diagnostic (Marburg, Germany). The principle of the method is based on the fact that the analyzed proteins form immune complexes with specific antibodies, which can scatter an incident light beam within an immunochemical reaction. The intensity of scattered light is proportional to the concentration of the analyzed protein in the sample. The “N Antisera to Human Apolipoprotein A-I)” assay (Siemens Healthcare Diagnostics Products GmbH, Marburg/Germany) was performed for ApoA1 determination, with an intra- and inter-assay coefficient of variation of 2.7 and 2.3%, respectively. For α-1 antitrypsin test, “The N Antisuera for Human α-1 antitrypsin” (Siemens Healthcare Diagnostics Products GmbH, Marburg/Germany) was performed, with intra- and inter-assay coefficients of variation of 2.7 and 1.9, respectively.

### 2.7. Statistical Analysis

Each categorical variable is expressed as a frequency (percentage) of cases. Continuous variables were tested for normal distribution by the Kolmogorov–Smirnov test. The normal distributed continuous variables are shown as mean ± SD, and those non-parametrically distributed are shown as median (interquartile range). Differences between groups were assessed by the unpaired Student *t* test or the Mann–Whitney U test for continuous variables and the ANOVA or Kruskal–Wallis test (as appropriate). Multivariate analysis by linear regression, adjusted by confounding factors such as age and gender, was used to identify the factors independently associated to biomarkers. For the concentrations of ApoA1 and α-1 antitrypsin, a receiver operating characteristic curve (ROC) was drawn, and the area under the curve was measured using a non-parametric test. The diagnostic performance of a test or the ability of a test to discriminate between drowning and other causes of death was evaluated using ROC curve analysis. The analysis of ROC curves provides an excellent view. We selected markers with areas below the curve greater than 0.70. The cut-off point was taken as the point nearest the ‘‘ideal’’ point of the ROC curve with highest sensitivity and lowest 1-specificity. For discriminant analysis, the ApoA1 cut-off level point was tested for its ability to distinguish between two diagnostic groups. A limitation of the method is that no multiple testing corrections can be applied. Statistical analyses were carried out with SPSS version 24.0 software for Windows (IBM SPSS Statistics, Inc., Chicago, IL, USA).

## 3. Results

### Proteomic Approach

We performed a pilot proteomic experiment with plasma samples from four forensic cases (50% men). A total of 343 proteins were identified from protein-depleted plasma samples (Table S1).

**Figure 1:**
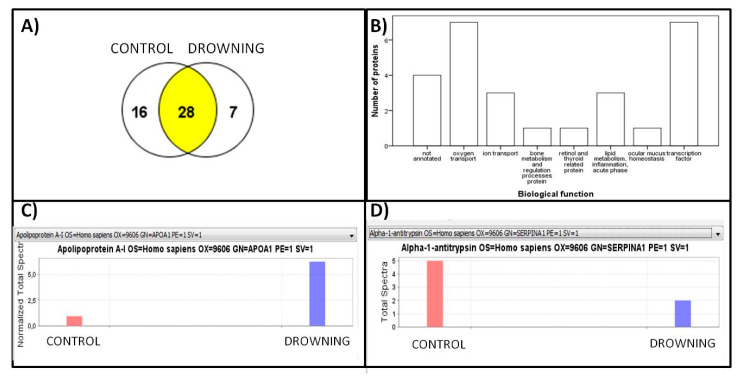
(**A**). Venn Diagram of proteins grouped by the cause of death (obtained from Scaffold viewer). (**B**). Number of proteins (grouped by their annotated biological function) commonly expressed in both drowning and control groups. Protein sizes and chromosome locations are available at https://www.uniprot.org/. (**C**) and (**D**) Quantitative view of normalization of differentially expressed ApoA1 and α-1 Antitrypsin, respectively (obtained from Scaffold viewer).

All samples were analyzed according to the cause of death: 2097 spectra were detected at an 88.0% threshold, with 0.43% decoy FDR; 51 proteins were identified at 99.0% threshold, with minimum 1 peptide and 6.2% decoy FDR. After sample quantification of these 51 proteins, 28 were common proteins, 16 proteins only appeared in the control group and 7 in deaths by drowning (Figure 1A). All the identified proteins were studied attending at their annotated biological function, relative expression pattern and published pathophysiological pathways. Figure 1B shows the number of proteins grouped by their biological functions. Apo A1 and α-1 antitrypsin resulted in being differentially expressed, and being less abundant in drowning, whereas apolipoprotein-A1 seemed to be more expressed in drowning (Figure 1C).

**Figure 2:**
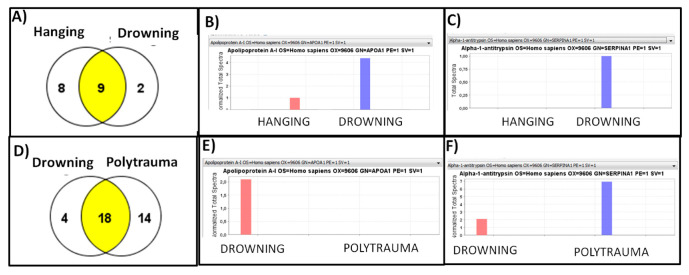
(**A**) Venn diagram of proteins in males case vs. control sub-analysis. (**B**) Quantitative view of normalization of differentially expressed ApoA1 in males case vs. control sub-analysis. (**C**) Quantitative view of normalization of differentially expressed α-1 Antitrypsin in males case vs. control sub-analysis. (**D**) Venn diagram of proteins in females case vs. control sub-analysis 1 and 6 were control cases. (**E**) Quantitative view of normalization of differentially expressed ApoA1 in females case vs. control sub-analysis. (**F**) Quantitative view of normalization of differentially expressed α-1 Antitrypsin in females case vs. control sub-analysis.

When performing sub-analyses as case vs. control samples, we analyzed a drowning case vs. a death by hanging, and 905 spectra were included at 99.0% threshold, with 0.12% decoy FDR; 19 proteins were identified at 99.0%, threshold, with minimum 1 peptide and 5.6% decoy FDR. After sample quantification, we observed nine shared proteins, eight in the death by hanging and only two in the drowning sample (Figure 2A). Again, apolipoprotein-A1 and α-1 antitrypsin appeared differentially expressed in drowning and death by hanging Figure 2B,C, but with lower levels of α-1 antitrypsin in hanging. On the other hand, when comparing drowning vs. polytrauma, 1325 spectra were detected at 83.0% threshold, with 0.80% decoy FDR; 37 proteins were identified at 99.0% threshold with 1 minimum peptides and 5.7% decoy FDR. Of these 37 proteins, 18 shared proteins were observed. 4 proteins were only identified in the drowned case and 14 in the case of death by car accident (Figure 2D). In this comparison, α-1 antitrypsin appeared more expressed in the polytraumatized sample (Figure 2E). In addition, ApoA1 resulted in being more expressed in the death by drowning (Figure 2F).

**Figure 3:**
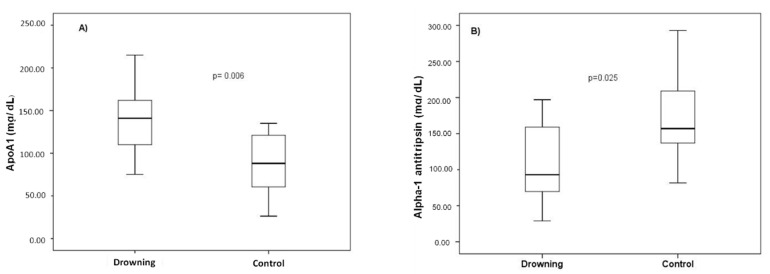
Plasma Apo A1 (**A**) and α-1 antitrypsin (**B**) levels comparison in drowning control samples (Student unpaired *T*-test).

For the validation experiment, we included 25 forensic cases, (18 (72%) men, 58.7 ± 18.2 years), within a postmortem interval of 23.0 ± 15.0 h. The postmortem interval exceeded 72 h only in one case of drowning. Quantitative data in drowning vs. control group for Apo A1 (141.32 ± 40.52 vs. 87.58 ± 38.37 mg/dL) and α-1 antitrypsin (108.73 ± 50.08 vs. 173.62 ± 75.76 mg/dL) were evaluated, and significant differences were obtained for both proteins (Figure 3). Statistical results were not significantly affected when recalculating excluding hanging control values, data not shown.

Now, we aimed to study whether the biomarkers were predictive for diagnosing death by drowning, and both ApoA1 (Table 1a) and α-1 antitrypsin (Table 1b) resulted in being associated with drowning. When adjusted by confounder factors, both remained as predictive independent factors (*p* = 0.010 and *p* = 0.022, respectively).

**Figure 4:**
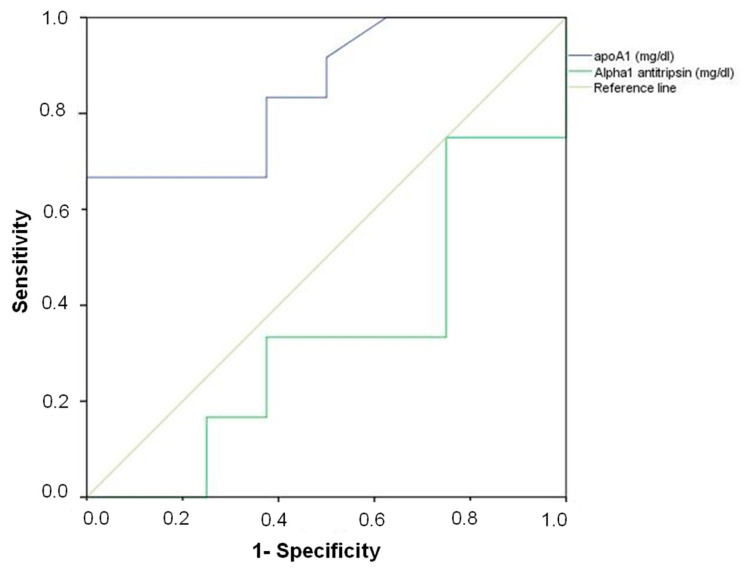
Receiver Operating Characteristic (ROC) curves of ApoA1 and α-1 antitrypsin levels in the prediction of death by drowning.

We constructed receiver operating characteristic (ROC) curves for biomarkers’ levels attending at the origin of death. When analysis was performed for drowning, ApoA1 levels showed an area under the curve of 0.85, *p* < 0.010, whereas α-1 antitrypsin values did not reach statistical significance (Figure 4, Table 2). ApoA1 cut-off point of 100 mg/dL, with the best specificity and sensitivity, was determined for drowning and selected (Table 2). A discriminant analysis was made with the cut-off showing the best discrimination curves between drowning and non-drowning. We used the diagnostic group as the grouping variable, establishing two groups: deaths from drowning and control cases. Correct classification using ApoA1 cut-off was reached for 73.9% of the cases (Table 2).

## 4. Discussion

Within the context of the forensic study of violent deaths, in some cases, it becomes difficult to determine an unequivocal diagnosis only based in the pathological findings. For this reason, an important field of clinical research has been developed in the search for complementary tests that facilitate postmortem diagnosis in these situations.

We performed a pilot proteomic experiment with plasma samples obtained from forensic cases. Since protein expression patterns may be influenced by such interpersonal demographic characteristics as age and sex [24,25] we selected paired samples. Hence, we used samples from 4 forensic cases: two drowning cases and two controls (one hanging, sharing pathophysiological asphyctic context with drowning, and one radically different kind of death, polytrauma). After applying restrictive parameters in the data processing for peptide and protein identifications, we selected proteins showing different expression levels in drowning and control samples. ApoA1 levels resulted in being more highly expressed in drowning when compared with control cases, whereas for α-1 antitrypsin, we found lower levels in those drowned, except for the case of death by hanging, according to the mentioned common etiology of death with drowning.

To better corroborate our results, we performed a validation experiment with plasma samples of drowned and controls. Data from the validation experiment revealed, for the first time, that both ApoA1 and α-1 antitrypsin are predictive independent factors for death by drowning, even after adjusting by confounder factors.

To our knowledge, neither of the two proposed biomarkers have been studied in the context of the pathophysiology of drowning before. Several studies have reported that patients rescued from drowning share clinical characteristics with those with acute respiratory distress syndrome, normally showing better evolution in drowning patients due to temporary and local injury [26]. Important roles for apolipoprotein A1 have been reported in the modulating pathogenesis of many respiratory diseases, such as attenuating inflammation, oxygen stress or bronchoalveolar lavage [27,28]. Recent investigations have indicated that Apo A1 is involved in several respiratory pathologies, such as neutrophilic airway inflammation, in type II asthma [29]. Other anti-inflammatory and immunomodulatory properties have been proposed for Apo A1, that may be relevant for adaptive immune responses in allergic asthma [30,31]. Hence, elevated contents of ApoA1 may be related to the bronchoalveolar lavage, inflammation and oxygen stress processes occurring in drowning.

Alpha 1 antitrypsin is a serine protease inhibitor which is largely produced by hepatocytes, but also produced by lung alveolar cells, monocytes and macrophages [32]. A relevant role in inflammation and infection processes in the lung has been proposed. α-1 antitrypsin deficiency has been associated with the destruction of alveolar walls and emphysematous changes due to elastase induced elastin breakdown in the lungs [32]. α-1 antitrypsin is involved in the protection of lung alveolar cells [33]. Interestingly, decreased levels of α-1 antitrypsin have been reported in asthma and bronchiectasis [34,35]. Therefore, the decreased level of α-1 antitrypsin showed in the plasma of our drowning cases may be explained for the presence of acute inflammation, oxygen stress and protease activity in their lungs. We also found lower levels of α-1 antitrypsin in the case of hanging, even when comparing with drowning. To date, the exact mechanism leading to death by hanging has yet to be elucidated. However, the principal patho-physiological theories are based on mechanisms of respiratory asphyxia, interruption to cerebral blood flow due to occlusion of vessels in the neck, and cardiac inhibition secondary to nerve stimulation [36]. Given that the pathophysiological pathways seem to be partially shared between the two asphyxias, it is not surprising that α-1 antitrypsin appeared down-expressed in both hanged and drowned cases.

Attending the patho-physiology of drowning and drowning survivals, the clinical effects of pulmonary edema, loss of surfactant, and increased permeability of the alveolar–capillary membrane resulted in decreased lung compliance, reduction of the perfusion in the lungs, atelectasis, and bronchospasm [37,38,39]. In fact, with both salted and fresh water, the hydrostatic forces over the alveolar-capillary membrane disrupted its integrity and plasma enters the alveoli, incapacitating normal gas exchange and generating foam. This resulted in decreasing pulmonary efficiency and respiratory distress. [40]. However, drowning physiology is complex and ongoing research in the underlying mechanisms of processes, such as fear of drowning, with sympathetic activation, diving response with oxygen conservation, autonomic conflict with the interplay between sympathetic and parasympathetic components of the nervous system upper airway reflexes, water aspiration and swallowing, may contribute to the prevention and survival of drowning [41].

The application of new technologies as -omics (genomic, transcriptomics, epigenetics/imprintomics, proteomic and metabolomics) promises to dramatically impact on forensic sciences [42]. Relevant information may help in the so-called “molecular autopsy”, as a complementary tool to the classical autopsy. However, studies already published in forensic proteomics are scarce, and mainly focused in the searching of an accurate PMI or the age estimate [17,43,44,45,46]. Furthermore, aside from some scarce investigations into cardiac deaths or sudden infant death syndrome, only few studies have been devoted to investigating the cause of death, in which using human forensic cases adds undeniable value [47,48,49].

The main limitation of our study is limited sample size, with only 16 cases in the drowning group and 9 in the control one, perhaps resulting in a loss of statistical significance in some of our analysis. In addition, corpses in an advanced state of decomposition were not included. Frequently, in real cases, in deaths by drowning, the subjects are found in an advanced state of decomposition. Our study is not valid to test how this event can influence our results. Furthermore, we have included cases with PMI < 72 h and demonstrated no influence of this parameter in the two biomarkers in our study; however, we cannot discard potential influence in future similar studies with larger cohorts and/or extended PMI. Despite all these limitations, statistically robust analyses with promising diagnostic implications have been achieved in this study.

We herein, and based on proteomic analysis, propose two biomarkers: apolipoprotein A1 and α-1 antitrypsin to contribute, together with forensic examinations, to improve the diagnosis of drowning. None of them have directly been related before with the patho-physiological context of drowning deaths. Moreover, we propose a cutoff point for ApoA1 of 100 mg/dL to help in the correct classification of deaths by drowning. Hence, our proteomic approach may result in a new complementary tool, by adding relevant information to the classical autopsy in the forensic diagnosis of drowning. Future research is assured to corroborate our results, as well as clinical implications in the personalized management of drowned patients.

## Figures and Tables

**Table 1:** Association analysis (linear regression) of Apo A1 (**a**) and α-1 antitrypsin (**b**) levels with death by drowning.

(**a**)					
**Dependent Variable**	**Unstandardized Coefficients**		**Standardized Coefficients**	**t**	***p*-Value**
**Univariate**	B	95%CI for B	β		
Drowning	−53.75	−90.0–(−17.49)	−0.56	−3.08	**0.006**
**Multivariate**					
Drowning	−54.69	−3.82–(−14.26)	−0.56	−2.84	**0.010**
Age	0.13	−0.96–(1.21)	0.05	0.24	0.811
Gender	−9.40	−49.89–31.18	−0.09	−0.48	0.633
(**b**)					
**Dependent Variable**	**Unstandardized Coefficients**		**Standardized Coefficients**	**t**	***p*-Value**
	B	95%CI for B	β		
Drowning	64.90	9.13–120.66	0.48	2.43	**0.025**
**Multivariate**					
Drowning	−68.54	−10.88–126.19	0.50	2.45	**0.022**
Age	−0.69	−2.36–0.98	−0.18	−0.87	0.395
Gender	30.62	−30.0–91.22	0.21	−1.06	0.303

B: Regression coefficient; 95%CI: 95% confidence interval. Statistically significant values appear in bold.

**Table 2:** Areas below the ROC curves, standard error and lower and upper limits of the area. Cut-off points established according to the use of a receiver operator characteristic curve. Discriminant analysis using ApoA1 in drowning.

Variable	Area	Standard Error	*p*-Value	Lower Limit	Upper Limit	Cut-Off Point	Sensitivity of Cut-Off Point	1-Specificity of Cut-Off Point	Drowning (% Correct Classification)	Non Drowning (% Correct Classification)	Total (% Correct Classification)
ApoA1	0.85	0.09	0.010	0.68	1.0	100	0.89	0.50	86.7	50.0	73.9
α1-antitrypsin	0.33	0.13	0.217	0.08	0.58						

## Supplementary Materials

The following are available online at https://www.mdpi.com/2075-4418/10/10/747/s1; Table S1 provides data for the 343 proteins identified from plasma samples in the proteomics experiment.
